# Multispecific Substrate Recognition in a Proton-Dependent Oligopeptide Transporter

**DOI:** 10.1016/j.str.2018.01.005

**Published:** 2018-03-06

**Authors:** Maria Martinez Molledo, Esben M. Quistgaard, Ali Flayhan, Joanna Pieprzyk, Christian Löw

**Affiliations:** 1 Centre for Structural Systems Biology (CSSB), DESY and European Molecular Biology Laboratory Hamburg, Notkestrasse 85, 22607 Hamburg, Germany; 2 Department of Medical Biochemistry and Biophysics, Karolinska Institutet, Scheeles väg 2, 17177 Stockholm, Sweden

**Keywords:** POT, PepT or PTR family, PepT1 and PepT2 homolog, major facilitator superfamily, MFS, transporter, membrane protein, X-ray crystallography

## Abstract

Proton-dependent oligopeptide transporters (POTs) are important for uptake of dietary di- and tripeptides in many organisms, and in humans are also involved in drug absorption. These transporters accept a wide range of substrates, but the structural basis for how different peptide side chains are accommodated has so far remained obscure. Twenty-eight peptides were screened for binding to PepT_St_ from *Streptococcus thermophilus*, and structures were determined of PepT_St_ in complex with four physicochemically diverse dipeptides, which bind with millimolar affinity: Ala-Leu, Phe-Ala, Ala-Gln, and Asp-Glu. The structures show that PepT_St_ can adapt to different peptide side chains through movement of binding site residues and water molecules, and that a good fit can be further aided by adjustment of the position of the peptide itself. Finally, structures were also determined in complex with adventitiously bound HEPES, polyethylene glycol, and phosphate molecules, which further underline the adaptability of the binding site.

## Highlights

• Dipeptides can adapt their position to best fit the PepT_St_ binding site
• The PepT_St_ binding site can adapt its structure to best fit the dipeptides
• The water network in the PepT_St_ binding site can adapt to best fit the dipeptides
• Aromatic binding site residues play a role in conferring PepT_St_ multispecificity

## Graphical Abstract

**Figure:**
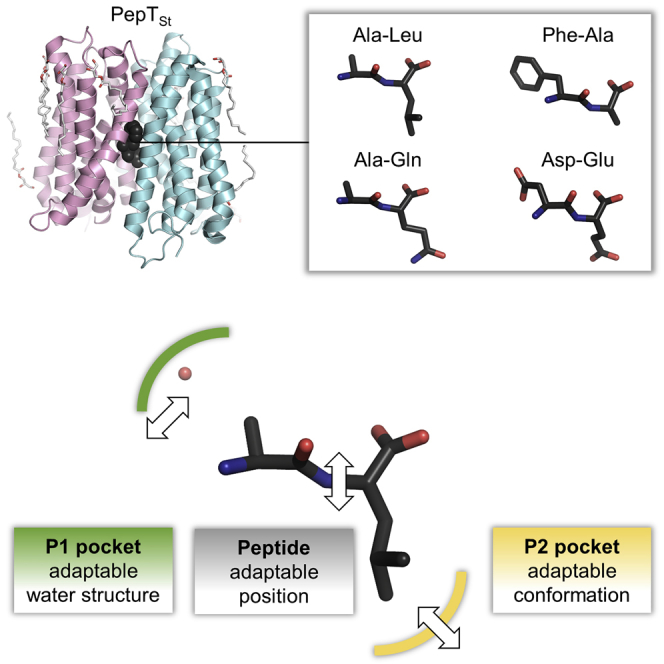

## Teaser

Proton-dependent oligopeptide transporters (POTs) are highly multispecific, accepting a broad range of substrates. Here, Martinez Molledo et al. characterize the substrate specificity of the POT transporter, PepT_St_. They present several structures of this protein in complex with different dipeptides and other ligands, revealing that multispecificity is achieved by several complementary strategies.

## Introduction

**Figure 1:**
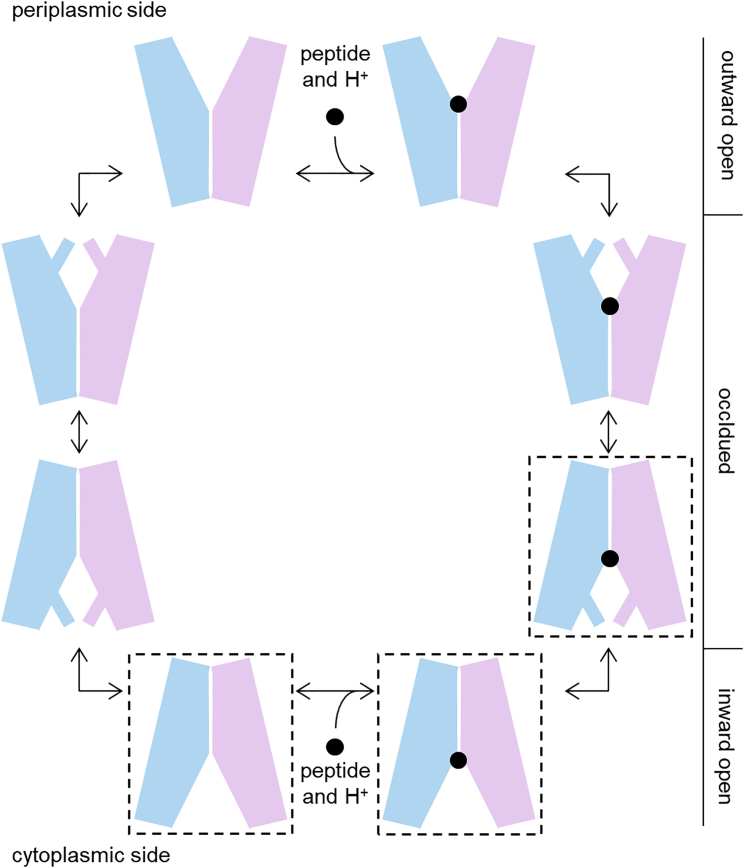
Functional Cycle of POTs The two MFS domains (N domain and C domain) are shown in light blue and pink, respectively. Similarly to other MFSs, POTs are believed to mediate substrate transport through alternating between an outward open state (binding site only accessible from the extracellular or periplasmic space) and an inward open state (binding site only accessible from the cytoplasm) via outward-facing and inward-facing occluded forms (access to the binding site restricted from both sides) (Quistgaard et al., 2016). The structures reported herein are all in one of the states highlighted by stippled boxes. From left to right, those are substrate-free inward open, substrate-bound inward open, and substrate-bound inward-facing occluded. Indeed all known POT structures conform to one of these three states.

Proton-dependent oligopeptide transporters (POTs; also known by the acronyms PepTs and PTRs) are ubiquitous in bacteria and eukaryotes where they play an important role in nitrogen acquisition through proton-coupled uptake of di- and tripeptides (Daniel et al., 2006, Smith et al., 2013). Furthermore, the human family members PepT1 and PepT2 also transport numerous peptidomimetic drugs and amino acid-conjugated prodrugs, and are thus of great pharmacological relevance (Brandsch et al., 2008, Smith et al., 2013). POTs belong to the major facilitator superfamily (MFS), and therefore adopt a canonical MFS fold where two 6-helical MFS domains, the N domain and the C domain, cradle a centrally located substrate binding cavity (Quistgaard et al., 2016, Yan, 2015). Two additional transmembrane helices are inserted between these domains in bacterial POTs (Newstead, 2015). The MFS fold enables substrate transport through an alternating access mechanism, which involves conformational transitions between inward open, outward open, and occluded states (Figure 1) (Newstead, 2015, Quistgaard et al., 2016, Yan, 2015). The substrate range of POTs is generally very wide; for example, human PepT1 and PepT2 can probably transport almost any di- and tripeptide (Brandsch et al., 2008). However, the preferences for different peptide side chains vary significantly among different POTs (Boggavarapu et al., 2015, Brandsch et al., 2008, Chiang et al., 2004, Ernst et al., 2009, Fang et al., 2000, Guettou et al., 2014, Ito et al., 2013, Sharma et al., 2016, Solcan et al., 2012), and some strongly prefer dipeptides to tripeptides (Boggavarapu et al., 2015, Ernst et al., 2009, Solcan et al., 2012). Structures have been determined for several different bacterial POTs in both apo and substrate-bound forms (Boggavarapu et al., 2015, Doki et al., 2013, Fowler et al., 2015, Guettou et al., 2013, Guettou et al., 2014, Lyons et al., 2014, Newstead et al., 2011, Quistgaard et al., 2017, Solcan et al., 2012, Zhao et al., 2014). Substrate-bound structures include GkPOT from *Geobacillus kaustophilus* in complex with the peptidomimetic alafosfalin (Doki et al., 2013), PepT_So2_ from *Shewanella oneidensis* in complex with alafosfalin, Ala-Ala-Ala, Ala-Tyr(Br), and Ala-Tyr(Br)-Ala (where Br indicates bromination) (Guettou et al., 2013, Guettou et al., 2014), and PepT_St_ from *Streptococcus thermophilus* in complex with Ala-Phe and Ala-Ala-Ala (Lyons et al., 2014). These structures have revealed that peptides generally bind across the binding cavity between the N domain and the C domain, though an alternative vertical binding mode was proposed for PepT_St_ in complex with Ala-Ala-Ala (Lyons et al., 2014). Yet our understanding of how multispecificity is achieved is still limited by the fact that the co-crystallized peptides exhibit very little sequence variation. In fact, as indicated above, they all consist of an N-terminal alanine residue followed by other alanine and/or aromatic residues.

Here, we set out to achieve a better understanding of the principles that govern multispecificity in POTs. Firstly, to characterize substrate preferences, we screened different peptides for *in vitro* binding to detergent-solubilized PepT_St_. Secondly, structures were obtained with Ala-Leu, Phe-Ala, Ala-Gln, and Asp-Glu in the binding site, as well as with various non-peptidic crystallant molecules, which were unexpectedly found to also bind here. These structures show how the binding site can adapt to peptide side chains with widely varying physicochemical properties, and thus shed significant new light on the structural basis for the broad substrate range of POTs.

## Results and Discussion

### Characterization of Substrate Specificity of PepT_St_

**Figure 2:**
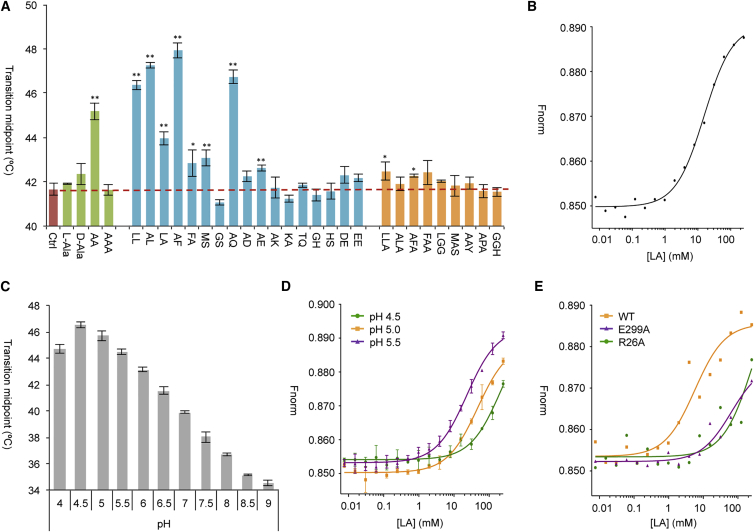
Peptide Binding Studies and Thermostability (A) Thermal stability studies using DSF. The peptide concentration was kept at 5 mM in all experiments. Green bars indicate peptides containing only alanine, blue bars indicate other dipeptides, and orange bars indicate tripeptides. The red bar and dashed horizontal line represent the control experiment with no substrate present. Error bars indicate the SD calculated from three independent measurements. The level of significant difference between the control and the rest of the samples is indicated by asterisks (^∗^p < 0.05 for significantly different samples and ^∗∗^p < 0.01 for highly significant differences). (B) Representative MST binding curve for Leu-Ala. (C) Effect of pH on thermostability as measured by DSF. Error bars indicate the SD calculated from three independent measurements. (D) Effect of pH on binding affinity as measured by MST. Binding curves are shown for binding of Leu-Ala to wild-type (WT) PepT_St_ at pH 4.5 (green), pH 5.0 (orange), and pH 5.5 (purple). Error bars indicate the SD calculated from three independent measurements. (E) Effect of selected mutations on binding affinity as measured by MST. Representative binding curves are shown for Leu-Ala binding to WT PepT_St_ (orange), E299A PepT_St_ (purple), and R26A PepT_St_ (green). These experiments were all carried out at pH 7.5.

The substrate specificity of PepT_St_ has been characterized previously using a proteoliposome-based competition uptake assay (Solcan et al., 2012). It was found that Ala-Ala is strongly preferred over Ala-Ala-Ala, and furthermore that hydrophobic dipeptides are better substrates than Glu-Glu and, in particular, Lys-Lys (Solcan et al., 2012). To complement these data, we used an expanded peptide library to test *in vitro* binding to detergent-solubilized PepT_St_ (Figure 2). Differential scanning fluorimetry (DSF) was used for initial screening of a library of 28 different peptides. The principle here is to measure the stabilization effect of the peptides on the transporter upon heat unfolding (Niesen et al., 2007). The most stabilizing peptides were found to be dipeptides with electroneutral side chains, while dipeptides with charged side chains and all tested tripeptides had either no or only little effect on thermostability (Figure 2A). For selected peptides, measurements were also carried out at various concentrations to affirm that the peptides are truly stabilizing (Figure S1). Next, we used microscale thermophoresis (MST) to quantify binding affinities of a subset of 17 peptides (Figure 2B and Table 1). These results revealed binding with low millimolar affinity of several different dipeptides, while all tested tripeptides exhibited very low affinity or no binding at all under the employed experimental conditions (Table 1). Concerning sequence specificity, it can be inferred that various small and large hydrophobic residues can be accommodated in both positions of dipeptide substrates, while charged residues appear to be generally disfavored (Table 1). Furthermore, Ala-Gln was also found to bind well, implying that a glutamine residue can be accommodated in the second position. Note that these conclusions are in line with both the DSF data (Figure 2A) and the previously published uptake results (Solcan et al., 2012).

**Table 1:** MST Results for Binding of Peptides to PepT_St_

Peptide	Protein	pH	Buffer	Salt	Detergent	K_D_ (mM)	±
Dipeptides							
Ala-Leu	WT	7.5	100 mM HEPES	150 mM NaCl	0.03% DDM	0.56	0.08
Ala-Phe	WT	7.5	100 mM Tris-HCl	150 mM NaCl	0.03% DDM	0.95	0.24
Leu-Leu	WT	7.5	100 mM Tris-HCl	150 mM NaCl	0.03% DDM	3.56	0.83
Ala-Gln	WT	7.5	100 mM Tris-HCl	150 mM NaCl	0.03% DDM	6.82	1.33
Ala-Ala	WT	7.5	100 mM Tris-HCl	150 mM NaCl	0.03% DDM	8.59	0.92
Phe-Ala	WT	7.5	100 mM Tris-HCl	150 mM NaCl	0.03% DDM	10.95	2.22
Leu-Ala	WT	7.5	100 mM HEPES	150 mM NaCl	0.03% DDM	13.34	0.87
Asp-Glu	WT	7.5	100 mM Tris-HCl	150 mM NaCl	0.03% DDM	>50	–
Ala-Asp	WT	7.5	100 mM Tris-HCl	150 mM NaCl	0.03% DDM	>100	–
Glu-Glu	WT	7.5	100 mM Tris-HCl	150 mM NaCl	0.03% DDM	–	–
Ala-Lys	WT	7.5	100 mM Tris-HCl	150 mM NaCl	0.03% DDM	–	–
Lys-Ala	WT	7.5	100 mM Tris-HCl	150 mM NaCl	0.03% DDM	–	–
Tripeptides							
Ala-Ala-Ala	WT	7.5	100 mM Tris-HCl	150 mM NaCl	0.03% DDM	>100	–
Ala-Pro-Ala	WT	7.5	100 mM Tris-HCl	150 mM NaCl	0.03% DDM	–	–
Leu-Leu-Ala	WT	7.5	100 mM Tris-HCl	150 mM NaCl	0.03% DDM	–	–
Ala-Phe-Ala	WT	7.5	100 mM Tris-HCl	150 mM NaCl	0.03% DDM	–	–
Ala-Leu-Ala	WT	7.5	100 mM Tris-HCl	150 mM NaCl	0.03% DDM	–	–
Screening of pH and Buffer Type							
Ala-Leu	WT	4.5	100 mM citrate	150 mM NaCl	0.03% DDM	>50	–
Leu-Ala	WT	4.5	100 mM citrate	150 mM NaCl	0.03% DDM	>100	–
Leu-Ala	WT	5.0	100 mM citrate	150 mM NaCl	0.03% DDM	47.05	5.11
Leu-Ala	WT	5.5	100 mM citrate	150 mM NaCl	0.03% DDM	23.04	1.87
Leu-Ala	WT	7.5	100 mM NaP_i_	150 mM NaCl	0.03% DDM	9.56	1.24
Leu-Ala	WT	7.5	100 mM Tris-HCl	150 mM NaCl	0.03% DDM	13.80	1.31
Screening of Binding Site Mutants							
Leu-Ala	R26A	7.5	100 mM Tris-HCl	150 mM NaCl	0.03% DDM	>50	–
Leu-Ala	E299A	7.5	100 mM NaP_i_	150 mM NaCl	0.03% DDM	>50	–
Leu-Ala	E300A	7.5	100 mM Tris-HCl	150 mM NaCl	0.03% DDM	–	–
Leu-Ala	E400A	7.5	100 mM NaP_i_	150 mM NaCl	0.03% DDM	–	–

A dash indicates that the binding was too weak for a K_D_ value (or K_D_ deviation) to be determined. DDM, n-dodecyl-β-D-maltoside.

### Effect of pH and Buffer System on PepT_St_ Stability and Ligand Binding

We next characterized PepT_St_ thermostability as a function of pH using DSF. Over a pH range of 4–9, stability was found to peak at a value of 4.5, after which it progressively decreased as the pH was further increased (Figure 2C). The influence of pH on substrate binding was then studied by measuring binding of Leu-Ala at pH 4.5, 5.0, 5.5, and 7.5. Here we found that binding affinities significantly drop as the pH is decreased from 5.5 (Figure 2D and Table 1). Thus, while acidic pH stabilizes the protein, it also weakens substrate binding. Finally, we found that changing the buffer from HEPES to Tris or phosphate (all at pH 7.5) has little or no discernible effect on affinity (Table 1).

### Structures of PepT_St_ in Complex with Dipeptides or without Substrate

**Figure 3:**
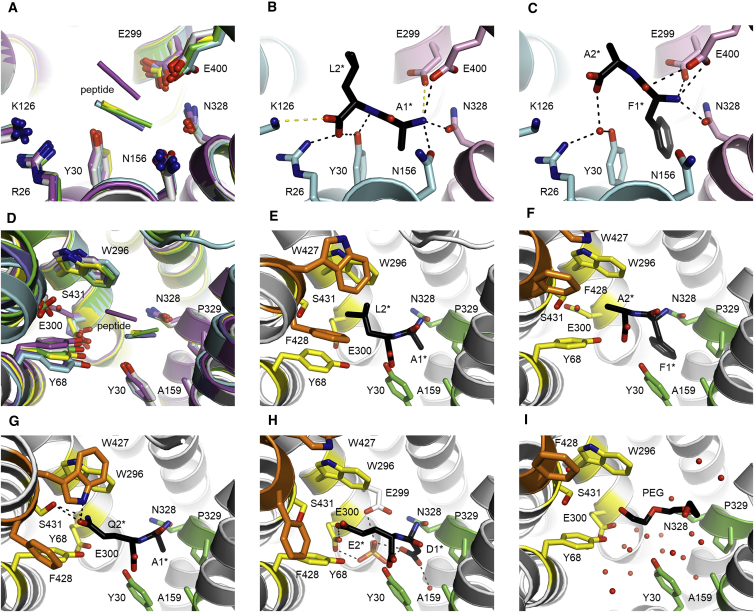
Structural Analysis of the Binding of Dipeptides to PepT_St_ (A–C) Main-chain interactions. (A) Overlay of PepT_St_[apo] (white), PepT_St_[Ala-Leu] (light blue), PepT_St_[Phe-Ala] (purple), PepT_St_[Ala-Gln] (green), PepT_St_[Asp-Glu] (yellow), and the previously published PepT_St_[Ala-Phe] (violet). Residues involved in binding of the peptide backbone are shown in stick representation. (B) Backbone interactions of Ala-Leu. Same orientation as in (A). The N domain is light blue and the C domain is pink. Black dashes indicate potential hydrogen bonds or salt bridges with lengths ≤3.2 Å, and yellow dashes indicate potential salt bridges with lengths of 3.2–4.0 Å. (C) Backbone interactions of Phe-Ala as shown in (B). (D–I) Side-chain interactions. (D) Same as in (A), except that the residues shown as sticks are the ones that form the P1 and P2 binding pockets, that the orientation is different, and that TM4 was truncated for clarity. (E) Binding of Ala-Leu. P1 is green, P2 is yellow, and P2-lid is orange. (F) Binding of Phe-Ala. (G) Binding of Ala-Gln. (H) Binding of Asp-Glu. (I) Solvent molecules in the binding cavity of the apo form (electron density map for the solvent is shown in Figure S3B). Water molecules are shown as red spheres and a putative PEG molecule, which we consider part of the solvent, is shown as sticks.

For crystallization we employed essentially the same crystallant as used for the previously published structures of PepT_St_ substrate complexes (Lyons et al., 2014), which contains HEPES, phosphate, and small polyethylene glycols (PEGs). The HEPES buffer was at pH 7.0, but the other ingredients of the crystallant decreased the overall pH of the system to between 5.0 and 5.8 depending on their concentration. Since binding is compromised at acidic pH, we also tried to obtain crystals at neutral pH or higher, but these attempts failed. The structures of PepT_St_ were determined in complex with four dipeptides that collectively represent substantial sequence variation: Ala-Leu, Phe-Ala, Ala-Gln, and Asp-Glu (Figure 3). These peptides, furthermore, bind with a rather wide range of affinities: high in the case of Ala-Leu (0.56 mM), intermediate for Ala-Gln (6.82 mM) and Phe-Ala (10.95 mM), and rather weak for Asp-Glu (>50 mM) (Table 1). The structures were refined to maximum resolutions of 2.7–2.2 Å (Table S1), and were either in the inward open conformation (PepT_St_[Phe-Ala], PepT_St_[Asp-Glu]) or in an inward-facing partially occluded form (PepT_St_[Ala-Leu], PepT_St_[Ala-Gln]), which mainly differs from the former by exhibiting pronounced bending of transmembrane helices 10 and 11 (TM10 and TM11), as described previously (Lyons et al., 2014, Quistgaard et al., 2017). The peptides were well defined in the electron density maps, with the exception of the glutamate side chain of Asp-Glu (Figures S2A–S2D). Obtaining a substrate/ligand free apo structure was not straightforward. Thus, when there was no peptide bound the binding site was not empty, but instead was occupied by non-peptidic molecules taken up from the crystallant, as described below. However, by changing the buffer to citrate pH 4.5, and thereby strongly inhibiting the binding, a high-quality 2.0-Å inward open apo structure could be obtained (Table S1).

### Interactions with the Dipeptide Backbone

An overlay of the structures reveals that the peptide backbones of Ala-Phe, Ala-Leu, Ala-Gln, and Asp-Glu overlap strongly with each other, while Phe-Ala adopts a markedly different position (Figure 3A). The backbone interactions of Ala-Leu, Ala-Gln, and Asp-Glu are similar to those previously described for Ala-Phe (Lyons et al., 2014): The N-terminal residue interacts with Asn-156, Glu-299, Asn-328, and Glu-400, while the C-terminal residue interacts with Arg-26, Tyr-30, and Lys-126 (Figure 3B). In the case of Phe-Ala, interactions with the PepT_St_ C domain are tighter, while the interactions with the N domain are either indirect (Arg-26, Tyr-30) or missing entirely (Lys-126, Asn-156) (Figure 3C). There are therefore no direct interactions with the C terminus of this peptide (Figure 3C). This may seem highly unfavorable, but it should be mentioned in this context that a mimic of the C-terminal carboxylate is not absolutely required for binding of peptidomimetic drugs to human PepT1 (Zhang et al., 2013). Notably, the residues interacting with the peptide backbone present only small variation in position regardless of whether a peptide is bound or not, and regardless of which orientation it adopts when bound (Figure 3A). Next, we tested the effect on binding of the dipeptide Leu-Ala when mutating Arg-26, Glu-299, Glu-300, or Glu-400 to alanine. As mentioned above, these residues are all seen to interact with the peptide termini in our structures, except for Glu-300. This residue may, however, be important for proton coupling, since substrate transport is adversely affected when it is mutated (Solcan et al., 2012), and since proton coupling in GkPOT was found to depend on the equivalent of this residue, Glu-310 (Doki et al., 2013). As expected, all these mutants exhibited either abolished or markedly reduced binding of the peptide (Figure 2E and Table 1).

### Binding Pockets for the Peptide Side Chains

The side chains of the peptides interact with two distinct pockets (Figures 3D–3I and surface views in Figures S2E–S2M): The first residue interacts with pocket 1 (P1), which is a quite hydrophobic groove lined by Tyr-30 and Ala-159 from the N domain, as well as Asn-328 and Pro-329 from the C domain, while the second residue interacts with pocket 2 (P2), which consists of Tyr-68, Trp-296, and Glu-300 from the N domain, and Ser-431 from the C domain. In addition, Trp-427 and Phe-428 from the flexible TM11 helix effectively form a lid on the P2 pocket (P2-lid) in the inward-facing partially occluded form (Figures 3E, 3G, and S2E–S2G), but not in the fully inward open structures (Figures 3F, 3H, and S2H–S2M). It is notable that the pockets are rich in aromatic residues, in particular P2 and P2-lid. Aromatic residues have the capacity to engage in not only hydrophobic interactions but also several specific arene interactions, such as arene-arene and arene-amide π-stacking, cation-π interactions, and X-H···π bonds (X = C, N, O) (Harder et al., 2013, Nishio et al., 2014, Salonen et al., 2011), which makes them ideally suited for multispecific recognition (Guettou et al., 2013, Ito et al., 2013, Pieri et al., 2009). Indeed, not only POTs but also, for example, multispecific drug transporters (Higgins, 2007) are characterized by having several aromatic residues in their binding sites. A comparison of all peptide complex structures reveals no significant variation in the positions of the P1 binding site residues, but substantial variation in P2 (Figure 3D). Of particular note, Tyr-68 adopts a range of different positions, which effectively tunes the size of the P2 pocket. Thus, in PepT_St_[apo] and PepT_St_[Phe-Ala] where there is either nothing or just a very small residue present in P2, the pocket is narrow, while in PepT_St_[Ala-Leu] and PepT_St_[Ala-Phe] where P2 instead harbors a bulky residue, the position of Tyr-68 is shifted to substantially widen it (Figure 3D). P1 and P2 are overall less well conserved than the residues interacting with the peptide backbone (Figures S2N–S2P), but this is not surprising considering that POTs have broad substrate preferences (Boggavarapu et al., 2015, Brandsch et al., 2008, Chiang et al., 2004, Ernst et al., 2009, Fang et al., 2000, Guettou et al., 2014, Ito et al., 2013, Sharma et al., 2016, Solcan et al., 2012). Tyr-68 is, however, very well conserved. Furthermore, mutating this residue to phenylalanine reduces the affinity for Glu-Glu (Solcan et al., 2012), and mutating the equivalent Tyr-64 in rabbit PepT1 to alanine abolishes transport activity (Chen et al., 2000). Tyr-68 is thus clearly functionally important, which might relate to its apparent role in tuning the P2 pocket.

### Interactions with Hydrophobic Side Chains

Ala-Leu and Phe-Ala are both hydrophobic, but differ in that Ala-Leu has a small N-terminal and a larger C-terminal residue, while the opposite is the case for Phe-Ala. Yet despite this, their side chains reach about equally deep into each of the two pockets (Figures 3E, 3F, and S2E–S2J). This is only possible because of their substantially different main-chain positions. We therefore hypothesize that the difference in overall position of these peptides (Figures 3A and 3D) is caused by the differently sized side chains pulling the backbone in diverging directions through optimizing their interactions with P1 and P2. A notable consequence of such an adaptation mechanism would be that binding of the residue in position 1 is not necessarily independent of the residue in position 2 and vice versa. Binding involves hydrophobic and van der Waals interactions as well as specific arene interactions (Figures 3E and 3F). The latter include arene-π and amide-π stacking interactions between the phenylalanine of Phe-Ala and Tyr-30 and Asn-328 in P1, respectively, as well as putative C-H···π interactions between the aliphatic side chains of the C-terminal peptide residues and Trp-296 in P2. As mentioned above, PepT_St_[Ala-Leu] is inward-facing partially occluded (Figure 3E). This conformation is stabilized by interactions between the peptide and P2-lid in a similar way as previously described for PepT_St_[Ala-Phe] (Lyons et al., 2014). A notable aspect of this binding mode is that the leucine side chain is hemmed in by several aromatic residues (Y68, W296, W427, and F428—see Figure 3E), which is reminiscent of the “aromatic clamp” model that has recently been proposed for the multidrug MFS transporter MdtM (Alegre et al., 2016). Interestingly, it thus appears that POTs and MFS multidrug transporters to some extent engage their substrates in similar ways. In PepT_St_[Phe-Ala], the alanine side chain of the peptide is on the other hand too small to aid in recruitment of P2-lid and thus in stabilization of the occluded form, which probably explains why this particular substrate complex crystallized in the inward open state. The stability of the substrate-bound inward-facing occluded form is thus apparently dependent on the exact nature of the bound substrate.

### Interactions with Polar and Acidic Side Chains

Ala-Gln binds in much the same position as Ala-Leu (Figures 3A and 3D). Yet the glutamine side chain is bound quite differently than leucine (Figure 3G): Tyr-68 and to a lesser extent Trp-296 have moved closer to it, thus narrowing the pocket around it and enabling the formation of van der Waals interactions as well as a putative N-H···π bond mediated by Trp-296 (Figure 3G). In addition, Trp-427 and Ser-431 have undergone rotameric shifts in order to form hydrogen bonds to the side-chain amide (Figure 3G). In PepT_St_[Asp-Glu], the aspartate Cβ atom forms similar van der Waals interactions with P1 as the alanine residues of Ala-Leu and Ala-Gln, while the carboxylic acid moiety is directed out of the groove. Here it forms a hydrogen bond with a well-ordered water molecule that is also conserved in the other structures, and potentially an additional hydrogen bond with a larger molecule, which we have interpreted as phosphate (Figures 3H and S2D). Unfortunately, the electron density for the glutamate side chain is rather poor and we therefore cannot conclude with any certainty how it binds in P2 (Figure S2D). Indeed, it is likely that it forms quite weak and flexible interactions here, in line with the rather low binding affinity of the peptide.

### Displacement of Ordered Water Molecules upon Substrate Binding

Due to the high resolution and good data quality, numerous ordered water molecules could be modeled in the binding cavity of PepT_St_[apo] (Figures S3A and S3B). These mostly reside at the apex of the cavity some distance away from where the peptide binds, but also occupy the center of the P1 groove (Figures 3I and S2K–S2M). In contrast, very few ordered water molecules are present in and around P2 (Figures 3I and S2K–S2M). In the substrate-bound structures, it was not possible to model quite as many water molecules. This is mostly due to somewhat lower resolution, but it is also clear that some of the water molecules found in the binding cavity of PepT_St_[apo] are incompatible with the presence of a peptide due to steric hindrance (Figures S3C–S3F). This suggests that solvation of the binding site might serve in facilitating substrate release, as has recently been suggested for the amino acid-polyamine-organocation transporter AdiC (Ilgu et al., 2016). Notably, which of the water molecules in PepT_St_[apo] that a given peptide would be incompatible with depends on the sequence of the peptide and especially on the nature of the N-terminal residue inserted into P1 (Figures S3C–S3F). Furthermore, while some water molecules are displaced by the peptide, others may instead facilitate its binding in a likewise sequence-dependent manner. Indeed, we have seen that the aspartate residue of the Asp-Glu peptide forms a hydrogen bond with one of the water molecules in P1, which is not formed in any of the other structures (Figures 3H and S3F). A general way of achieving multispecific binding in P1 may thus be to displace or retain specific water molecules to optimally match the pocket to the physicochemical properties of the incoming peptide side chain. This would be similar to what has been observed in OppA, which is a multispecific periplasmic binding protein that delivers peptide substrates to the ATP-binding cassette translocator, OppBCDF (Tame et al., 1994, Tame et al., 1996).

### Structures with Vertically Bound HEPES

**Figure 4:**
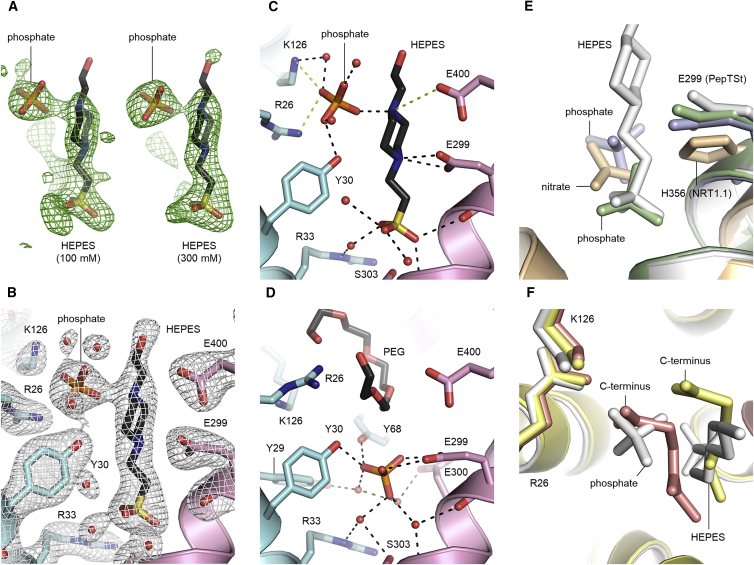
Binding of Non-peptidic Molecules (A) Evidence for binding of HEPES and phosphate. The modeled HEPES and phosphate molecules in PepT_St_[100 mM HEPES] (left) and PepT_St_[300 mM HEPES] (right) are shown together with their F_o_-F_c_ difference density omit maps contoured at 3σ. (B) Refined electron density map for HEPES and the co-bound phosphate molecule. The 2F_o_-F_c_ map for PepT_St_[HEPES 300 mM] is contoured at 1σ. (C) Interaction mode of HEPES and the co-bound phosphate molecule. HEPES, phosphate, and interacting residues are shown as sticks, and water molecules involved in binding are shown as red spheres. Hydrogen bonds and salt bridges are shown as in Figure 3. (D) Interaction mode of phosphate in PepT_St_[phosphate], depicted as in (C) and in the same orientation. For electron density map and interactions of the PEG molecule, see Figure S5. (E) Upper acid/anion binding region. An overlay is shown of PepT_St_[HEPES 300 mM] (white), PepT_St_[phosphate] (pale violet), PepT_St_[Asp-Glu] (pale green), and AtNRT1.1 (PDB: 4OH3 [Sun et al., 2014]) (wheat). (F) Lower acid/anion binding region. An overlay is shown of PepT_St_[HEPES 300 mM] (white), PepT_St_[Ala-Leu] (salmon), and PepT_St_[Phe-Ala] (pale yellow).

When co-crystallizing PepT_St_ with some substrates, we observed an elongated blob of positive difference density flanked by a smaller roundish one (Figures 4A and S4A–S4C), which is not congruent with the substrate being bound in a horizontal manner between the two MFS domains similarly to Ala-Leu, Phe-Ala, Ala-Gln, and Asp-Glu. We initially thought that the elongated vertical blob would represent the previously proposed vertical peptide binding mode (Lyons et al., 2014). However, the density looked essentially the same for a variety of substrates with very different compositions and dimensions, including Ala-Tyr, Thr-Gln, Ala-Ala-Ala, Val-Tyr-Val, and valganciclovir (Figures S4A–S4C), and it therefore became clear that it almost certainly does not represent the substrate, but more likely a molecule that was present in the crystallant in all cases. The only such molecule that could fit into the electron density is HEPES (Figures 4A and S4). A structure was therefore refined with HEPES in the vertical difference density blob and phosphate in the flanking position (Table S1). Next, we tested increasing concentrations of HEPES from 100 mM to 300 mM and obtained a 2.2-Å structure (Table S1) with better electron density for the HEPES molecule (Figures 4A, 4B, and S4A). On the other hand, when crystallizing with citrate or phosphate buffer instead of HEPES, the vertical difference density blob was absent. Finally, we found that HEPES could also be modeled in two previously published structures that were obtained using the same crystallization conditions as employed in this study: PDB: 4D2D where Ala-Ala-Ala had originally been modeled, and PDB: 4D2B where no ligand had been modeled (Figures S4D–S4G). This finding suggests that the previously proposed vertical binding mode for Ala-Ala-Ala should perhaps be re-evaluated. The HEPES molecule is bound as follows. The sulfonate group is located at the apex of the binding cavity where it interacts with several water molecules and the backbone of the kinked TM7 helix (Figure 4C), while the two nitrogen atoms of the HEPES ring interact with Glu-299 and potentially Glu-400, which are also used for binding the N terminus of dipeptide substrates (Figure 4C). The flanking phosphate molecule is paired with HEPES through an interaction with the nitrogen atom farthest away from the sulfonate group. Apart from that, it also interacts with Arg-26 and Lys-126 similarly to the C terminus of Ala-Leu, as well as with several water molecules (Figure 4C). It is thus clear that the HEPES-phosphate pair mimics some aspects of how peptides are bound despite not having a peptide-like composition.

### Upper and Lower Acid/Anion Binding Sites

When crystallizing PepT_St_ using phosphate buffer, the binding site was occupied by a phosphate and a PEG molecule. A structure was refined to 2.4 Å maximum resolution, which displayed excellent electron density for both non-peptidic ligands (Figures S5A and S5B). The PEG molecule interacts with Arg-26 and P2/P2-lid (Figures S5C and S5D), causing the protein to adopt an occluded conformation that differs somewhat from the occluded forms obtained with peptides, indicating that the inward-facing occluded form is not a very well defined state (Figure S5E). Interestingly, the phosphate molecule does not bind in the same place as in the structure with HEPES and phosphate, but is instead localized in the water-filled apex of the binding cavity (Figure 4D). Here it forms a double hydrogen bond with Glu-299, which must thus be protonated, and additional hydrogen bonds with Tyr-30 and Tyr-68 as well as several water molecules (Figure 4D). It follows that acids/anions can bind in two different regions. We have termed the one that is close to the apex of the cavity and the extracellular side the “upper region” (Figure 4E), and the one that accommodates the C terminus of dipeptides the “lower region” (Figure 4F). In the upper region, the HEPES sulfonate group overlaps strongly with the phosphate molecule modeled in PepT_St_[Asp-Glu] but only partially with the one found in PepT_St_[phosphate] (Figure 4E). In the lower region, we find a strong overlap of the phosphate paired with HEPES and the C terminus of Ala-Leu. The HEPES ring overlaps, however, not with Ala-Leu but with the backbone of the phenylalanine of Phe-Ala (Figure 4F). POTs are closely related to NRT1 nitrate transporters (Tsay et al., 2007). It is therefore intriguing that the nitrate binding site of AtNRT1.1 (Parker and Newstead, 2014, Sun et al., 2014) coincides with the upper acid/anion binding region identified here for PepT_St_ (Figure 4E). In fact, the key nitrate binding residue in AtNRT1.1, His-356, is directly equivalent to the key phosphate binding residue in PepT_St_, Glu-299 (Figure 4E). This prompts the question of whether binding of acids/anions in the upper region might also be biologically relevant in PepT_St_ and/or other POTs. However, while this would be interesting to investigate further, it lies beyond the scope of the present study.

### Principles Enabling Multispecificity in POTs

We have previously postulated that multispecificity in POTs arises in part because the majority of the hydrogen bonds and salt bridges involved in binding of peptide substrates engage the universal backbone structure rather than the variable side chains (Guettou et al., 2013). We show here that in addition to this the P1 and P2 side-chain binding pockets can adapt to diverse peptide substrates in at least two different ways: modulation of the water network (observed for P1) and movement of binding site residues (observed for P2, but possibly also applicable to P1 under some conditions). In addition, the substrate can also itself adapt to the binding pockets by moving between partially different positions. The structures, furthermore, underline the significance of the high prevalence of aromatic residues in the binding sites of POTs, as these exhibit considerable variability in position and in how they engage the substrates. Finally, our data suggest that the previously described vertically bound tripeptide may have been a HEPES molecule that was misidentified due to low occupancy and moderate resolution.

## STAR★Methods

### Key Resources Table

**Table:** 

REAGENT or RESOURCE	SOURCE	IDENTIFIER
**Chemicals**, **Peptides**, **and Recombinant Proteins**		

Vector for protein expression pNIC-CTHF-PepT_St_	Löw et al., 2013, Quistgaard et al., 2017	N/A
Protein expression and purification protocol	Löw et al., 2013, Quistgaard et al., 2017	N/A
L-Ala	Fluka	Cat# 5129
D-Ala	Aldrich	Cat# 162655
Ala-Ala	Sigma	Cat# A502
Ala-Ala-Ala	Sigma	Cat# A9627
Leu-Leu	Bachem	Cat# M-1535
Ala-Leu	Sigma	Cat# A1878
Leu-Ala	Bachem	Cat# G-2460
Ala-Phe	Sigma	Cat# A3128
Phe-Ala	Bachem	Cat# G-2850
Met-Ser	Sigma	Cat# M9380
Gly-Ser	Sigma	Cat# G3127
Ala-Gln	Sigma	Cat# G8541
Ala-Asp	Bachem	Cat# G-1195
Ala-Glu	Bachem	Cat# G-1200
Ala-Lys	Bachem	Cat# G-1290
Lys-Ala	Bachem	Cat# G-2630
Thr-Gln	Sigma	Cat# T3275
Gly-His	Sigma	Cat# G1627
His-Ser	Sigma	Cat# H3129
Asp-Glu	Sigma	Cat# A1916
Glu-Glu	Sigma	Cat# G3640
Leu-Leu-Ala	Bachem	Cat# H-3905
Ala-Leu-Ala	Bachem	Cat# H-5975
Ala-Phe-Ala	Bachem	Cat# H-5420
Phe-Ala-Ala	GL Biochem	N/A
Leu-Gly-Gly	Sigma	Cat# L9750
Met-Ala-Ser	Sigma	Cat# M1004
Ala-Ala-Tyr	Bachem	Cat# H-1445
Ala-Pro-Ala	Bachem	Cat# H-1595
Gly-Gly-His	Sigma	Cat# G4541
n-nonyl- β-D-maltoside (NM) detergent	Anatrace	Cat# N330
n-dodecyl-β-D-maltoside (DDM) detergent	Anatrace	Cat# D310
1-(7Z-pentadecenoyl)-rac-glycerol (7.8 MAG)	Avanti Polar Lipids, Inc.	Cat# 850531O

**Deposited Data**		

Crystal structure of PepT_St_[Ala-Leu]	This paper	PDB: 5OXL
Crystal structure of PepT_St_[Ala-Gln]	This paper	PDB: 5OXK
Crystal structure of PepT_St_[Asp-Glu]	This paper	PDB: 5OXM
Crystal structure of PepT_St_[Phe-Ala]	This paper	PDB: 5OXN
Crystal structure of PepT_St_[100 mM HEPES]	This paper	PDB: 6EIA
Crystal structure of PepT_St_[300 mM HEPES]	This paper	PDB: 5OXQ
Crystal structure of PepT_St_[phosphate]	This paper	PDB: 5OXP
Crystal structure of PepT_St_[apo]	This paper	PDB: 5OXO

**Software and Algorithms**		

XDS / XSCALE / XDSCONV	(Kabsch, 2010)	
PHENIX	(Adams et al., 2010)	
PHASER	(McCoy et al., 2007)	
COOT	(Emsley et al., 2010)	
MolProbity	(Chen et al., 2010)	
Consurf		http://consurf.tau.ac.il/2016/
PyMol		Schrödinger LLC; http://www.pymol.org
Protein Data Bank (PDB)		www.pdb.org
GraphPad Prism		https://www.graphpad.com

### Contact for Reagent and Resource Sharing

Further information and requests for resources and reagents should be directed to and will be fulfilled by the Lead Contact, Christian Löw (christian.loew@embl-hamburg.de).

### Method Details

#### Protein Expression and Purification

For WT PepT_St_, we used the same construct as reported previously (Löw et al., 2013). Full-length cDNA of PepT_St_ wild type (WT) was previously amplified from the *Streptococcus thermophilus* genome, and cloned into a pNIC-CTHF vector by ligation-independent cloning (LIC). This vector contains a C-terminal His-Tag and a Tobacco Etch virus (TEV) cleavage site. The vector carries a kanamycin resistance gene as selectable marker. For generating the additional single mutants presented in this study, either blunt-end polymerase chain reaction (PCR) or QuikChange PCR was used.

The constructs were expressed in *E*. *coli* C41 cells. For protein expression, cells were grown in terrific broth (TB) media supplemented with 30 μg/ml kanamycin. Cultures were grown at 37°C and protein expression was induced with 0.2 mM IPTG at an OD_600_ of 0.6 - 0.8. After induction, culture growth continued at 18°C for 16-18 hours. Cells were harvested by centrifugation (7000 rpm, 15 minutes, 4°C in JLA 8.1 rotor of Avanti JXN-26 centrifuge, Beckman Coulter), and the pellet was stored at -20°C until further use.

Purification of WT and mutant PepT_St_ were carried out as previously described (Löw et al., 2013, Quistgaard et al., 2017). In brief, the cell pellet was resuspended in lysis buffer (20 mM NaP_i_ at pH 7.5, 300 mM NaCl, 5% (v/ v) glycerol, 15 mM imidazole, 5 ml of lysis buffer per gram of wet weight pellet), supplemented with lysozyme (1 mg/ml final concentration), DNase (5 units/ml) and 0.5 mM tris(2-carboxyethyl)phosphine (TCEP). The cells were lysed by three cycles using an EmulsiFlex-C3 (Avestin) at 10.000-15.000 psi. Recovered material was centrifuged to remove non-lysed cells (7000 rpm, 15 minutes, 4°C in JLA 8.1 rotor of Avanti J-20 XP centrifuge) and the supernatant was subjected to ultracentrifugation to separate the membrane fraction (35000 rpm, 1 hour, 4°C in 45 Ti rotor of Optima XE-90 centrifuge, Beckman Coulter). Membranes were resuspended in lysis buffer supplemented with protease inhibitors (one tablet per 100 ml lysis buffer, Roche), and solubilized by adding 1% n-dodecyl-β-D-maltoside (DDM) detergent. Solubilized PepT_St_ was firstly purified by immobilised-metal affinity chromatography (IMAC) on a gravity column. The beads were pre-equilibrated in lysis buffer supplemented with 0.03% DDM and 0.5 mM TCEP and incubated with the solubilized membrane proteins for one hour at 4°C on a rotating wheel. Loaded beads were extensively washed with wash buffer with increasing imidazole concentrations (20 mM NaP_i_ at pH 7.5, 300 mM NaCl, 5% glycerol, 15-30 mM imidazole, 0.5 mM TCEP, 0.03% DDM). The protein was eluted from the column with a buffer containing high imidazole concentration (20 mM NaP_i_ at pH 7.5, 150 mM NaCl, 5% glycerol, 250 mM imidazole, 0.5mM TCEP, 0.03% DDM) and combined with 1 ml of TEV protease at 1 mg/ml to perform the His-tag cleavage during dialysis overnight at 4°C. Typically, 1 mg of TEV protease was sufficient to cleave the purified protein from 3 liters of culture. The dialysis buffer contained 20 mM Tris-HCl at pH 7.5 (pH adjusted at room temperature), 150 mM NaCl, 5% glycerol, 0.5 mM TCEP, 0.03% DDM. Cleavage was successful > 90%, and the cleaved protein was recovered by negative IMAC. A second purification step was done by size-exclusion chromatography (SEC). The cleaved protein was concentrated to 5 ml using a 100 kDa concentrator (Corning® Spin-X® UF concentrators) and run on an ÄKTA Pure system (GE Healthcare Life Sciences), using a HiLoad 16/ 600 Superdex 200 column (GE Healthcare Life Sciences). Fractions containing the protein were pooled and concentrated to 10 mg/ml, flash frozen and stored at -80°C until further use.

PepT_St_ WT was also purified in different detergents for the DSF studies. In this case, the purification procedure was identical to the one described previously with the exception of the detergent added to each buffer. In the purification with n-nonyl-β-D-maltoside (NM), the membrane solubilisation was performed by adding 1% n-decyl-β-D-maltopyranoside (DM) and the buffers used for the IMAC purification were supplemented with 0.1% DM. In the SEC step, the gel filtration buffer contained 0.4% NM. As the micelle size in this last case is smaller, the concentration was performed using 50 kDa concentrators (Corning® Spin-X® UF concentrators). As before, the protein was flash frozen and stored at -80°C until further use.

#### Peptide Stock Preparation

Peptides were purchased from Sigma-Aldrich, Bachem and GL Biochem (Shanghai). Peptide stocks were prepared by weighing the lyophilized powder using an analytical balance and diluting them in ultrapure water at the highest possible concentration. The approach published by Huang et al. was used for estimating the solubility of the peptides (Huang et al., 2012).

#### DSF Measurements

The transition midpoint (Tm) for thermal unfolding of PepT_St_ was determined with the nanoDSF Prometheus NT.48 devise (NanoTemper technologies). The change in fluorescence at 330 nm and 350 nm was recorded over a temperature ramp of 20 – 90°C with an increase of one degree per minute. For each run, we used ∼10 μl protein at a concentration of 0.5 mg/ml. For this experiment, PepT_St_ was solubilized in n-nonyl-β-D-maltoside (NM), as the effect of peptide addition was significantly higher in short-chain detergents than using the markedly more stabilizing n-dodecyl-β-D-maltoside (DDM) (Quistgaard et al., 2017). In the initial screen, the final peptide concentration was 5 mM (Figure 2A), but for a selected group of peptides, measurements were also carried out at additional concentrations in the range of 1.25 – 20 mM (Figure S1). The unfolding curves were plotted using GraphPad Prism (GraphPad Software, San Diego, CA). A Student’s t-test was performed to determine significant differences between the control sample and those in presence of a peptide at 5 mM.

#### MST Measurements

PepT_St_ peptide binding was measured with the Monolith NT.LabelFree microscale thermophoresis device (NanoTemper technologies). The principle here is that ligand binding induces changes in the mobility of the target protein in a temperature gradient, which can be detected by monitoring intrinsic tryptophan fluorescence originating from PepT_St_ (Seidel et al., 2013). For each binding experiment, 16 different samples were prepared in which the protein concentration was kept constant (125 nM), whereas the peptide was added in a concentration series up to the millimolar range. Measurements were performed in the final buffer concentrations indicated in Table 1. For the pH-dependent measurements, the peptides were dissolved in the corresponding buffer. For the other measurements, the peptide stocks were prepared in water. Measurements were performed at 22°C, 15-20% LED power and 20% or 40% MST power. Binding curves were plotted and analyzed using GraphPad Prism.

#### Crystallization

All structures reported in this paper were obtained from crystals grown using the lipidic cubic phase (LCP) method (Aherne et al., 2012). The same protocol described by Lyons et al. was used to form the mesophase (Lyons et al., 2014). In brief, PepT_St_ protein (10 mg/ml) was mixed with lipid 1-(7Z-pentadecenoyl)-rac-glycerol (MAG 7.8, Avanti Lipids) in a one to one volume ratio, using two coupled Hamilton syringes. Crystallization plates were set up using the Mosquito-LCP robot (TTP Labtech). The used protocol dispensed 50 nl of mesophase in the wells of one row of the plate and straight after, the mesophase was covered with 800 nl of precipitant solution. The plates used for crystallization were the Laminex™ UV Plastic Bases with wells of 100 μm depth (Molecular Dimensions), which were manually sealed with Laminex™ UV Plastic 200 micron Film Covers (Molecular Dimensions). Different strategies were tested to obtain complexes of PepT_St_ purified in DDM and the peptides of interest. Ala-Leu and Phe-Ala were added to the screen in concentrations of 5 mM and 100 mM, respectively. Ala-Gln was premixed with PepT_St_, reaching a final concentration of 100 mM, and once the mesophase was produced, it was incubated at room temperature for one hour. For Asp-Glu, a dry coating approach was used (Gelin et al., 2015): Each well of the crystallization plate was coated with 1 μl of 10 mM Asp-Glu, and the drops were then left drying for two days at room temperature before setting up the screen. The screen used for crystallization was based on the condition described previously (Lyons et al., 2014), and thus contained 0.1 M HEPES buffer at pH 7.0, 0.15-0.55 M NH_4_H_2_PO_4_ and 15-25% PEG 400. However, HEPES was replaced with different buffers for growing crystals of PepT_St_[apo] and PepT_St_[phosphate]. Specifically, crystals of the former grew in 0.1 M citrate buffer pH 4.5 and 15-30% PEG 300, while crystals of the latter grew in 0.1 M NaP_i_ buffer pH 6.5, 250 mM NH_4_H_2_PO_4_ and 15-30% PEG 400. Crystals generally appeared within 24 hours and continued growing the following 3 days, and were harvested and flash frozen in liquid nitrogen within 7-10 days.

#### Data Collection and Structure Determination

Crystal screening and data collection were done at the EMBL P13 and P14 beamlines at the PETRA III storage ring (c/o DESY, Hamburg, Germany) and ID30A and B at the European Synchrotron Radiation Facility (ESRF, Grenoble, France). All structures were determined from a single crystal, except for PepT_St_[Phe-Ala] were three data sets were merged. The data were indexed, reduced and scaled using the XDS program suite (Kabsch, 2010) (Table S1). The maximum resolution at which to cut the data was decided based on completeness and the CC1/2 correlation coefficient promoted by Karplus and Diederich (Karplus and Diederichs, 2012, Karplus and Diederichs, 2015). Regardless of the buffer system used, the crystals consistently adopted the same crystal form as previously described by Lyons et al. (Lyons et al., 2014) (Table S1). An initial model was placed using the Phaser molecular replacement program from the PHENIX suite (Adams et al., 2010, McCoy et al., 2007). Refinement was then carried out through several cycles of manual rebuilding in Coot and maximum likelihood refinement in PHENIX version 1.9_1692, where translation libration screw (TLS) was used (Adams et al., 2010). The final models were validated using MolProbity (Chen et al., 2010). ConSurf was used for analyzing sequence conservation and mapping it on the structures (http://consurf.tau.ac.il/2016/) (Ashkenazy et al., 2016). In total, 150 sequences that are 35 – 85% identical to PepT_St_ were used in this analysis. All omit maps presented in this study were generated by repeating the last round of refinement after removal of the omitted substrate/ligand from the input PDB file. PyMol was employed for generating structural overlays and making structure figures (Schrödinger LLC; http://www.pymol.org).

### Quantification and Statistical Analysis

The statistical test performed for this study was the Student’s t-test to measure the level of significant different between the control sample and PepT_St_ in the presence of different peptides in the DSF measurements. For this purpose, GraphPad Prism (GraphPad Software, San Diego, CA) was used. For the DSF and MST data, the indicated values represent the average of three independent measurements and the standard deviation calculated from them.

### Data and Software Availability

#### PDB Accession Numbers

Coordinates and structure factors for the PepT_St_ structures presented herein have been deposited in the protein data bank (PDB) with the following accession numbers: 5OXK (PepT_St_[Ala-Gln]), 5OXL (PepT_St_[Ala-Leu]), 5OXM (PepT_St_[Asp-Glu]), 5OXN (PepT_St_[Phe-Ala]), 5OXO (PepT_St_[apo]), 5OXP (PepT_St_[phosphate]) 5OXQ (PepT_St_[HEPES 300 mM]) and 6EIA (PepT_St_[HEPES 100 mM]).

## Supplemental Information

Document S1. Figures S1–S5 and Table S1

Document S2. Article plus Supplemental Information
