# Supplementary material for: Multispecific Substrate Recognition in a Proton-Dependent Oligopeptide Transporter
Source: Structure. 2018 Mar 6;26(3):467–476.e4. doi: 10.1016/j.str.2018.01.005 (PMC5845931; doi:10.1016/j.str.2018.01.005)
Supplement: Document S1. Figures S1–S5 and Table S1 [file mmc1.pdf]

Structure, Volume *26*

Supplemental Information

Multispecific Substrate Recognition in a Proton-Dependent Oligopeptide Transporter

Maria Martinez Molledo, Esben M. Quistgaard, Ali Flayhan, Joanna Pieprzyk, and Christian Löw

$PepT_{St}$ complex with:	Ala-Leu	Ala-Gln	Asp-Glu	Phe-Ala	HEPES-100mM	HEPES-300mM	Phosphate	Apo
**Data collection**								
Beamline	ESRF ID30A-1	PETRA III P14	ESRF ID30B	PETRA III P14	PETRA III P13	PETRA III P13	PETRA III P13	PETRA III P13
Wavelength (Å)	0.9660	0.9762	1.0396	0.9763	0.9796	0.9763	0.9763	1.0332
Space group	$C222_1$	$C222_1$	$C222_1$	$C222_1$	$C222_1$	$C222_1$	$C222_1$	$C222_1$
Cell dimensions								
a, *b*, *c* (Å)	102.30, 110.60, 108.50	100.70, 110.20 104.20	100.60, 109.00, 107.00	100.80, 107.90 109.80	102.49, 110.03, 110.56	102.21, 110.05, 109.50	101.60, 110.10, 107.90	102.10, 110.30, 110.70
α, β, γ (°)	90, 90, 90	90, 90, 90	90, 90, 90	90, 90, 90	90, 90, 90	90, 90, 90	90, 90, 90	90, 90, 90
Resolution (Å)	46.27 – 2.66 (2.75 – 2.66)	48.81 – 2.38 (2.47 – 2.38)	48.56 – 2.30 (2.38 – 2.30)	45.81 – 2.20 (2.28 – 2.20)	49.25 – 2.00 (2.072 – 2.00)	46.31 – 2.19 (2.27 – 2.19)	49.04 – 2.37 (2.46 – 2.37)	49.36 – 1.95 (2.02 – 1.95)
R_{merge}	0.108 (0.594)	0.107 (1.121)	0.119 (1.439)	0.147 (1.411)	0.0651 (1.139)	0.132 (2.175)	0.094 (1.423)	0.122 (1.673)
$I/\sigma I$	9.58 (1.97)	18.75 (2.56)	10.33 (1.03)	14.61 (2.13)	23.25 (2.33)	14.90 (0.95)	14.09 (1.17)	16.20 (1.30)
CC1/2	0.994 (0.72)	0.999 (0.832)	0.998 (0.509)	0.997 (0.712)	0.999 (0.766)	1.000 (0.408)	0.999 (0.477)	0.999 (0.592)
Completeness (%)	98.2 (96.4)	99.7 (99.9)	99.0 (95.1)	99.5 (97.7)	99.8 (99.52)	99.7 (97.4)	99.6 (97.3)	99.6 (99.3)
Total no. reflections	53978 (5215)	314880 (31241)	172471 (17919)	568946 (41559)	556226 (55100)	415514 (37484)	162105 (15327)	603439 (60307)
Multiplicity	3.0 (2.9)	13.2 (13.4)	6.5 (6.8)	18.4 (13.9)	13.1 (13.2)	13.1 (12.0)	6.5 (6.4)	13.2 (13.5)
Wilson *B*-factor ($Å^2$)	49.82	47.07	53.66	47.21	41.15	45.45	58.04	37.29
**Refinement**								
R_{work} / R_{free}	0.224 / 0.233	0.208 / 0.222	0.215 / 0.235	0.194 / 0.214	0.181 / 0.200	0.190 / 0.205	0.194 / 0.208	0.181 / 0.197
No. atoms								
Protein	3463	3380	3394	3580	3629	3519	3572	3546
Ligands/ions (binding site)	14	15	23	17	20	20	21	10
Ligands/ions (elsewhere)	57	31	15	11	43	41	33	27
Lipids	220	286	352	264	396	396	352	440
Water	32	47	29	73	122	98	37	177
B-factors								
Protein	54.6	58.4	69.1	56.6	50.1	57.6	67.1	41.6
Ligands/ions (binding site)	47.0	88.3	110.0	85.8	82.0	73.9	76.9	81.0
Ligands/ions (elsewhere)	91.7	92.6	118.2	90.9	101.4	100.4	123.1	81.4
Lipids	74.8	89.5	92.8	84.5	84.4	87.2	92.8	78.9
Water	51.3	56.0	58.3	55.8	51.5	53.8	57.2	45.8
R.m.s. deviations								
Bond lengths (Å)	0.003	0.004	0.005	0.005	0.003	0.010	0.009	0.005
Angles (°)	0.755	0.833	0.916	0.804	0.779	1.135	1.135	0.877
Ramachandran								
Favored (%)	98.2	98.6	98.6	98.9	99.3	98.7	98.7	98.9
Outliers (%)	0.0	0.0	0.0	0.0	0.0	0.0	0.0	0.0
Clash score	3.4	3.0	6.7	4.7	3.1	5.4	5.9	4.0
PDB accession	5OXL	5OXK	5OXM	5OXN	6EIA	5OXQ	5OXP	5OXO

Table S1: Crystallographic data processing and refinement statistics. Related to Figures 3 and 4. For the data collection statistics, values in parentheses refer to the outer shell.

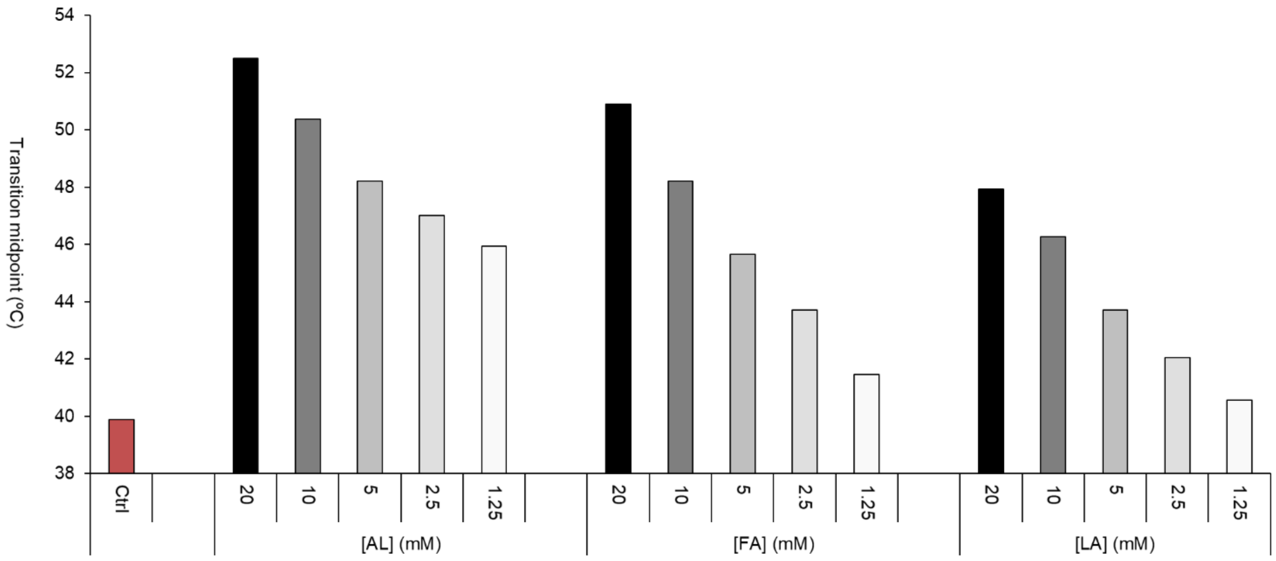

Figure S1. Concentration dependent DSF measurements for three selected peptides: Ala-Leu, Phe-Ala and Leu-Ala. Related to Figure 2. Measurements are colored grey/black with darker colors signifying higher peptide concentrations. The red bar represents the control experiment with no added peptide.

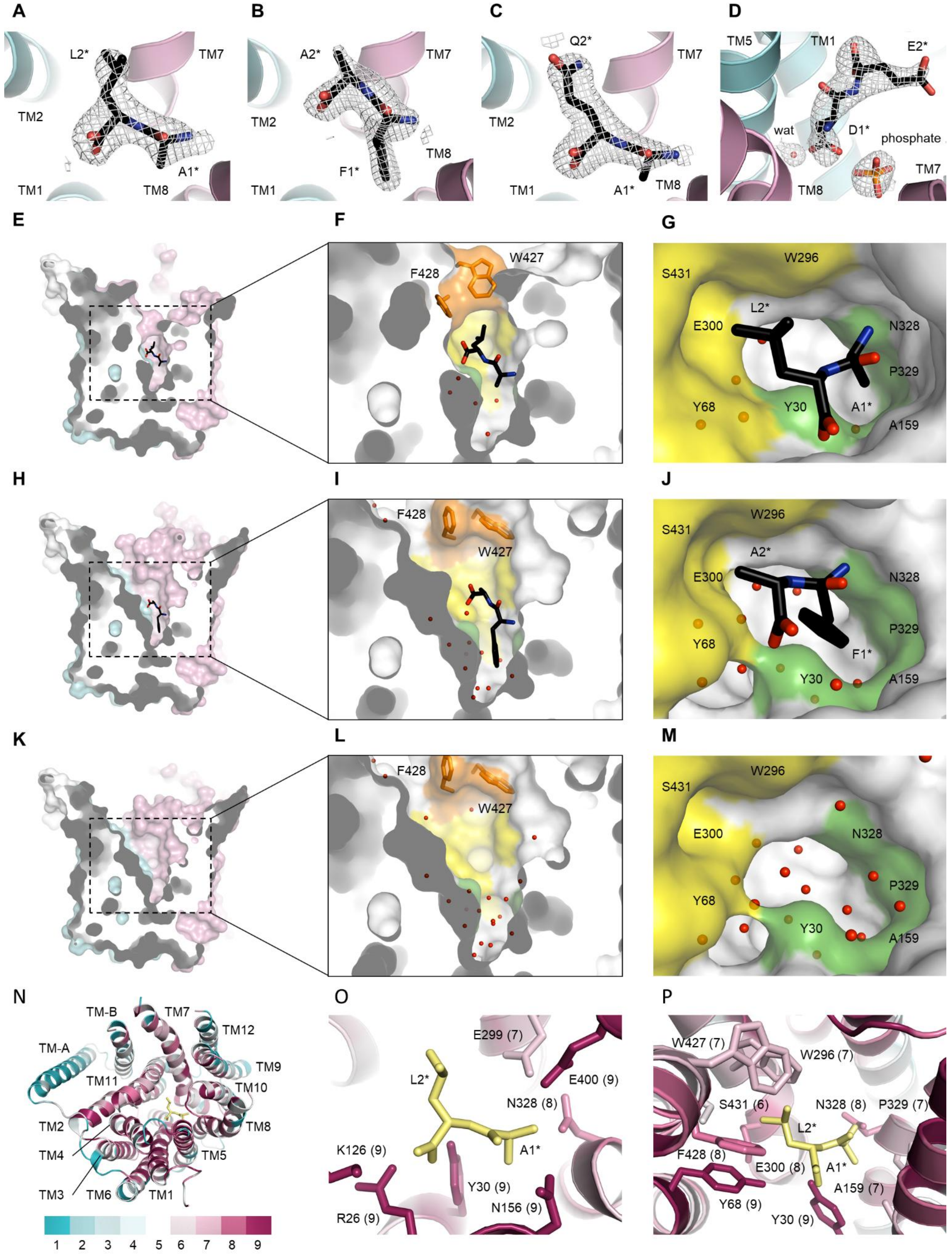

A
L2*
TM7
TM2
TM1
TM8
A1*
B
A2*
F1*
C
Q2*
D
TM5
E2*
wat
D1*
phosphate
E
F
F428
W427
G
S431
W296
E300
N328
P329
Y68
Y30
A159
H
I
J
K
L
M
N
TM-B
TM12
TM-A
TM9
TM11
TM10
TM4
TM3
TM6
1
2
3
4
5
6
7
8
9
O
E299 (7)
E400 (9)
N328 (8)
K126 (9)
Y30 (9)
N156 (9)
R26 (9)
P
W427 (7)
W296 (7)
S431 (6)
P329 (7)
F428 (8)
E300 (8)
A159 (7)
Y68 (9)

Figure S2. Extended analysis of the binding of dipeptides to $PepT_{St}$. Related to Figure 3. (A-D) Electron density maps for the bound dipeptides. N-domain is light blue, C-domain is pink, the peptides are black, and the 1-σ 2Fo-Fc electron density maps are grey. Transmembrane helices (TM) and peptide residues are labeled. (A) Binding of Ala-Leu. (B) Binding of Phe-Ala. (C) Binding of Ala-Gln. (D) Binding of Asp-Glu. This structure is shown in a different orientation than used for panels A-C in order to better show an interacting water molecule and a putative interacting phosphate molecule. The rotamers of the peptide side chains are generally fairly well defined in the electron density maps. An exception is the glutamate side chain of Asp-Glu. Here the general direction of the side chain is clear, but a different rotamer similar to the one adopted by the glutamine side chain in Ala-Gln could also fit fairly well. Notably, the electron density map is of poor quality not only for the glutamate side chain, but also for TM11, suggesting that the P2/P2-lid pocket is somewhat structurally heterogeneous in $PepT_{St}$[Asp-Glu]. (E-M) Surface views of the peptide binding site. (E) Side view of the binding site of $PepT_{St}$[Ala-Leu]. The protein is shown from the side in semitransparent surface representation and a part has been cut away to reveal the binding site in the middle of the protein. Colored as in panels A-D. (F) Zoomed side view of the binding site of $PepT_{St}$[Ala-Leu]. Colored as in Figure 3E-I: P1 is green, P2 is yellow and P2-lid is orange. The water molecules of the binding cavity are shown as small red spheres, and the two aromatic residues of P2-lid are shown, not only in semitransparent surface representation, but also as sticks. (G) Cytoplasmic view of the binding site of $PepT_{St}$[Ala-Leu]. P2-lid was omitted to allow an unobscured view of the peptide. (H-J) same as panels E-G, but for $PepT_{St}$[Phe-Ala]. (K-M) same as panels E-G, but for $PepT_{St}$[apo]. The PEG molecule shown in Figure 3I was omitted for clarity. (N-P) Sequence conservation, as analyzed using ConSurf (see methods). As indicated by the inset, the color of the protein is ramped from teal (low conservation, score = 1) over white to burgundy (high conservation, score = 9). The peptide is pale yellow. (N) Cytoplasmic view of $PepT_{St}$[Ala-Leu]. (O) View of the binding site residues interacting with the peptide backbone. Same orientation as in panel N, but zoomed in on the peptide. To ease interpretation, the conservation score

for each residue is given in parenthesis. (P) View of the binding site residues interacting with the peptide side chains, *i.e.* residues forming P1, P2 and P2-lid. Same orientation as in Figure 3E. Note that the binding site residues interacting with the peptide backbone are generally better conserved than those interacting solely with the peptide side chains.

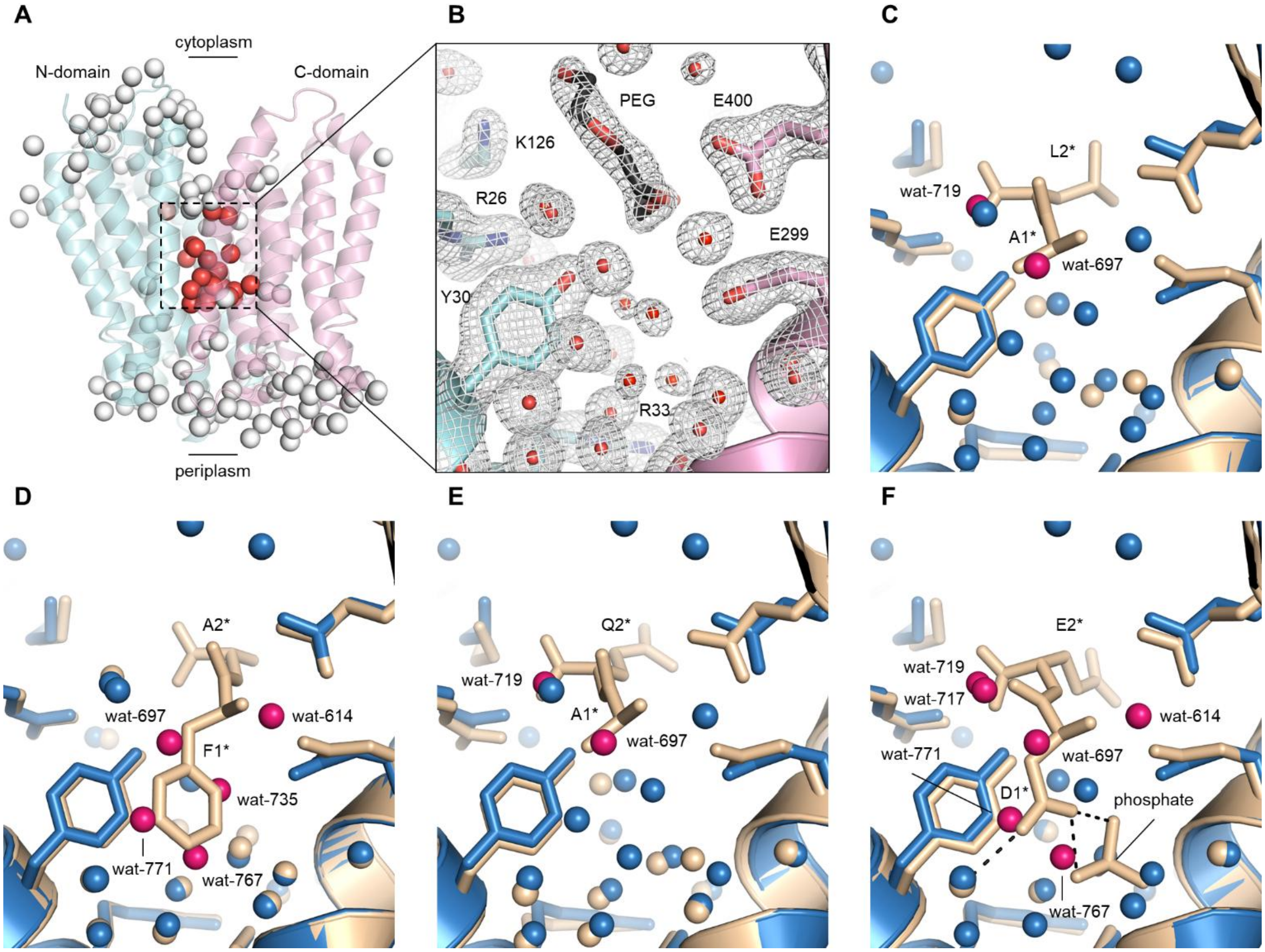

Figure S3. Extended analysis of the ordered solvent. Related to Figure 3. (A) All water molecules in PepT$_{St}$[apo]. N-domain is light blue, C-domain is pink and the water molecules are either white or red depending on whether they are also visible in panel B (red) or not (white). (B) Solvent molecules in the binding cavity of PepT$_{St}$[apo]. This represents a zoomed and clipped view of panel A, as indicated. It is the same view as used for Figure 4B–4D. Binding site residues and a modeled PEG molecule are shown as sticks along with the 1-σ 2Fo-Fc electron density map. The PEG molecule was modeled in a featureless somewhat twisted electron density blob. It may represent either a small intact PEG molecule or a part of a larger not fully ordered one. Other molecules that could also fit in this blob apart from PEG include the aliphatic tails of lipid or detergent molecules, but the lack of a hydrophobic

environment around it makes PEG a much more likely candidate. Since the putative PEG molecule forms no direct interactions with the protein and does not extend into either of the peptide side chain binding pockets (Figure 3I), it is probably best viewed as part of the solvent. (C) Comparison of water structure in $PepT_{St}$[apo] (blue) and $PepT_{St}$[Ala-Leu] (wheat). Same view as in panel B. Water molecules in $PepT_{St}$[apo], which would clash with the peptide, as it is bound in $PepT_{St}$[Ala-Leu], are highlighted in pink and labeled. In annotating these molecules, we used the criteria that they should be less than 2 Å removed from the peptide. (D) Comparison of water structure in $PepT_{St}$[apo] and $PepT_{St}$[Phe-Ala]. (E) Comparison of water structure in $PepT_{St}$[apo] and $PepT_{St}$[Ala-Gln]. (F) Comparison of water structure in $PepT_{St}$[apo] and $PepT_{St}$[Asp-Glu]. It is clear that some of the water molecules in $PepT_{St}$[apo] are incompatible with the presence of a peptide. This includes wat-697 and an additional set, which varies depending on the peptide sequence.

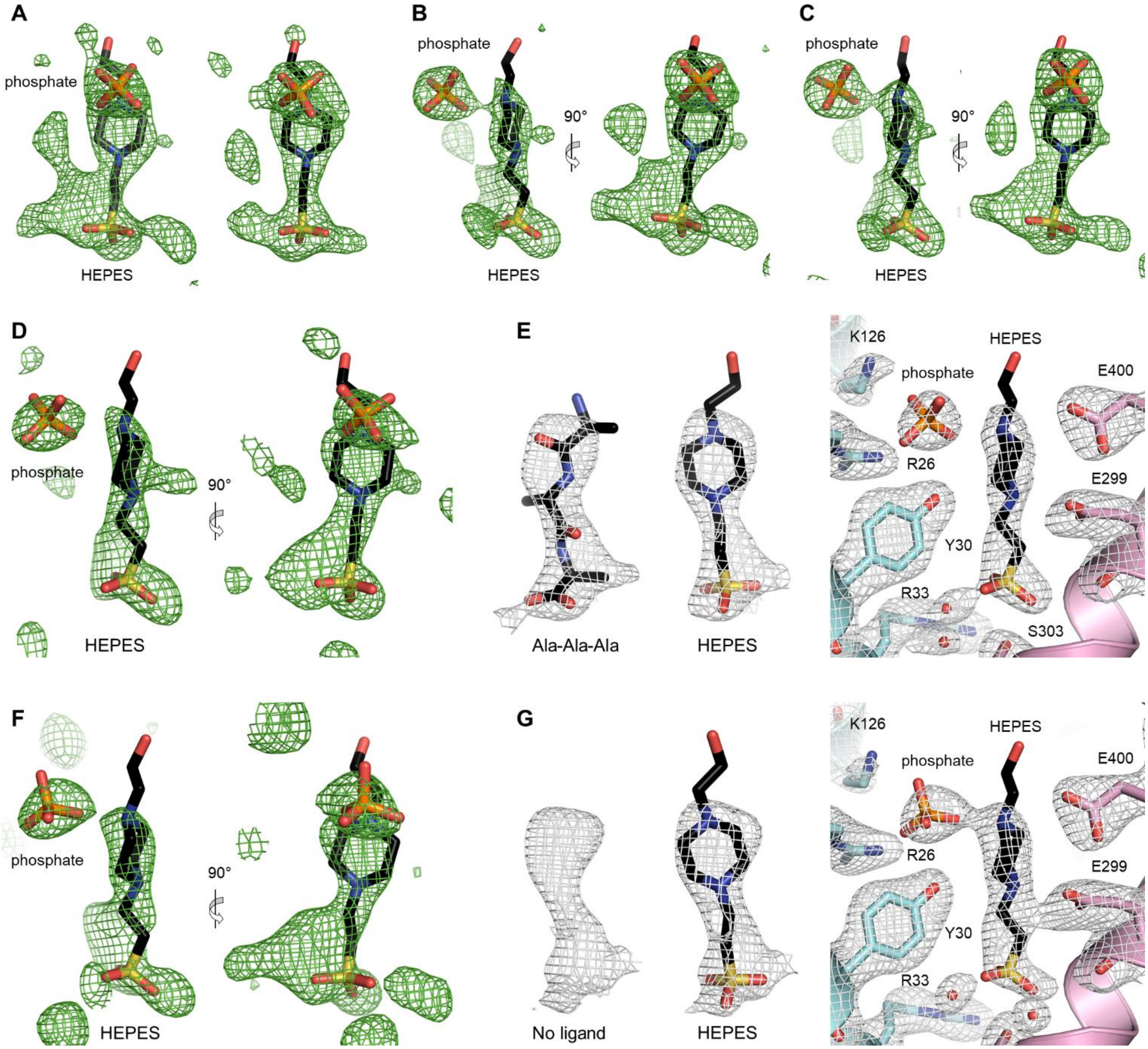

Figure S4. Extended analysis of the dual binding of HEPES and a phosphate molecule to PepT$_{St}$. Related to Figure 4. (A) 3-σ 1Fo-Fc omit maps for HEPES and phosphate are shown for PepT$_{St}$[100 mM HEPES] (left) and PepT$_{St}$[300 mM HEPES] (right). Same as Figure 4A, but viewing angle rotated 90 degrees. In the case of PepT$_{St}$[100 mM HEPES], HEPES fits fairly well in the electron density, but does not fill it out. This could be due to alternative conformations or to competition with other molecules that may be present with low occupancy in overlapping positions. In the case of PepT$_{St}$[300 mM HEPES], much of this extra difference density has disappeared, which aligns well with the latter

hypothesis. (B) 3-σ 1Fo-Fc omit map of HEPES and phosphate for $PepT_{St}$ co-crystallized with Val-Tyr-Val. (C) 3-σ 1Fo-Fc omit map of HEPES and phosphate for $PepT_{St}$ co-crystallized with Ala-Ala-Ala. Note that the maps are very similar regardless of whether Val-Tyr-Val, Ala-Ala-Ala or Ala-Tyr were used for co-crystallization (Ala-Tyr was present in the crystals used to obtain the $PepT_{St}$[100 mM HEPES] structure). Indeed, all structures we obtained using crystallants containing HEPES and phosphate presented either a horizontally oriented electron density blob corresponding to a dipeptide (Figure S2A-D) or similar blobs of electron density as shown here for Val-Tyr-Val, Ala-Ala-Ala and Ala-Tyr, thus affirming that these likely represent HEPES and phosphate. (D) Putative binding of HEPES and phosphate in re-refined PDB:4D2D. This structure was originally refined with a vertically bound Ala-Ala-Ala peptide, but was here re-refined with HEPES and phosphate. The 3-σ 1Fo-Fc omit map is shown. Note that this map is quite similar to what we have seen for Ala-Tyr, Val-Tyr-Val and Ala-Ala-Ala, and it is therefore very plausible that HEPES and phosphate are bound rather than Ala-Ala-Ala. (E) 1-σ 2Fo-Fc maps for re-refined PDB:4D2D with Ala-Ala-Ala left in place (left), and with Ala-Ala-Ala replaced with HEPES and phosphate (middle and right). HEPES and phosphate fit fairly well in the 2Fo-Fc map. Indeed, the HEPES piperazine ring matches better the shape of the electron density map here than does a peptide backbone. (F) Putative binding of HEPES and phosphate in re-refined PDB:4D2B. This structure was originally refined as ligand-free, but was here re-refined with HEPES and phosphate. While the vertical difference electron density blob was originally quite weak, it became significantly stronger after re-refining the model with guidance from the higher resolution structures presented in this work. The re-refined 3-σ 1Fo-Fc omit map thus suggests that the binding site is not unoccupied, and that HEPES and phosphate could potentially fit. (G) 1-σ 2Fo-Fc maps for re-refined PDB:4D2B with no ligands present (left), and with HEPES and phosphate added (middle and right). HEPES and phosphate fit rather well in the map.

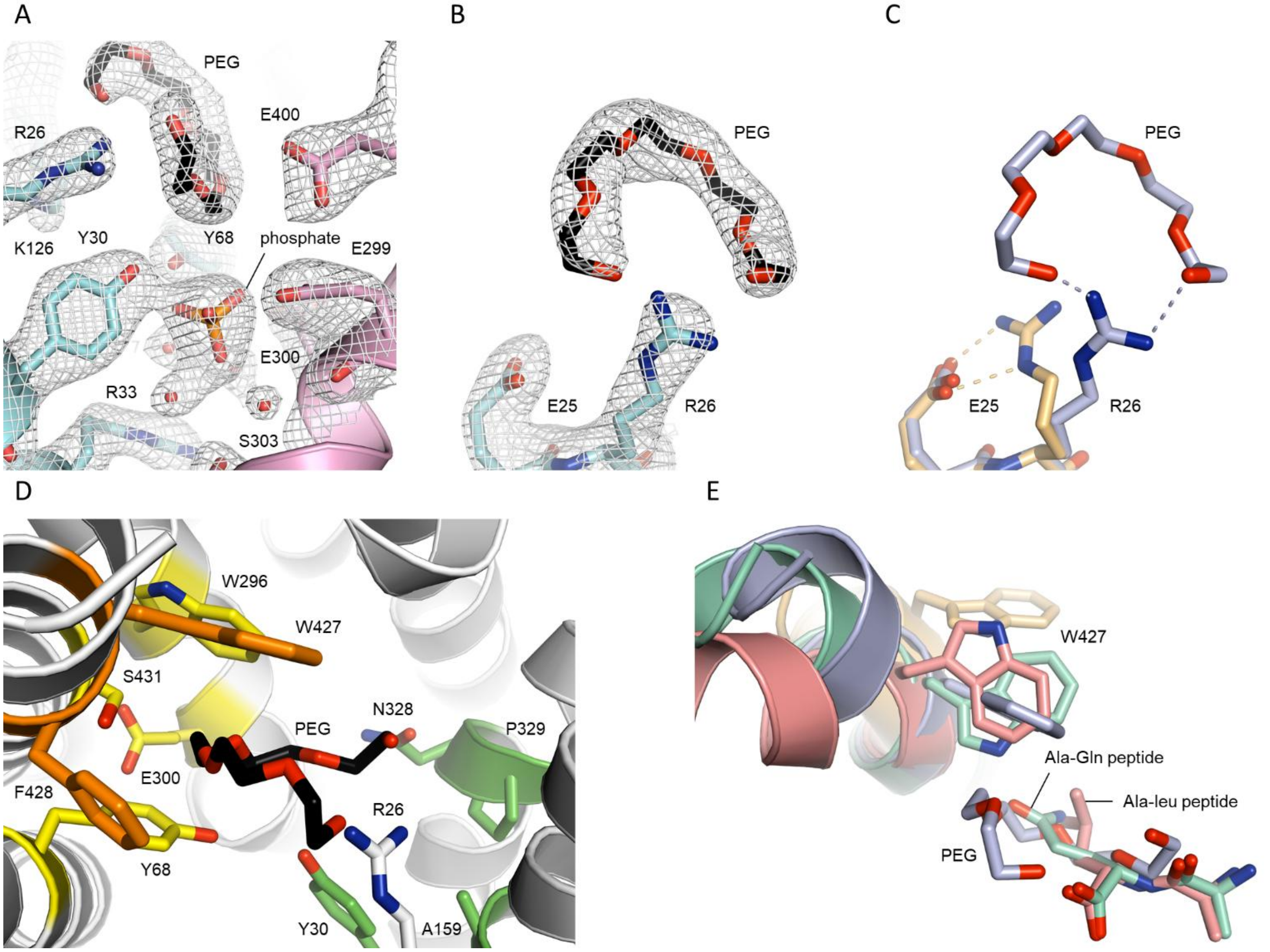

Figure S5. Extended analysis of PepT$_{St}$[phosphate]. Related to Figure 4. (A) Electron density map for the binding cavity. Shown as in Figure 4D except that the hydrogen bonds are omitted, and the 1-σ 2Fo-Fc electron density map is included. (B) Electron density map for the bound PEG molecule. The bound PEG molecule as well as Glu-25 and Arg-26 are shown along with the 1-σ 2Fo-Fc electron density map. (C) Repositioning of the side chain of Arg-26 and its interactions with the bound PEG molecule. Same view as in panel B, but here PepT$_{St}$[apo] (light orange) was overlayered on PepT$_{St}$[phosphate] (pale violet), and putative hydrogen bonds mediated by Arg-26 are shown as dashes, which follow the same color code as the protein. Arg-26 forms a double hydrogen bond/salt bridge with Glu-25 in PepT$_{St}$[apo] and indeed all other PepT$_{St}$ structures apart from PepT$_{St}$[phosphate]. However, in PepT$_{St}$[phosphate], it has undergone a rotameric shift, which has caused it to detach from

Glu-25 and move within hydrogen bonding distance of both tips of the PEG molecule. (D) Interactions of the bound PEG molecule with P2 and P2-lid. Same orientation and color scheme as in Figure 3E-I: P1 is green, P2 is yellow, P2-lid is orange, and PEG is black. The PEG molecule inserts deeply into P2/P2-lid where it packs against the faces of the aromatic rings of Tyr-68, Tyr-296, Trp-427 and Phe-428, thus resulting in the formation of numerous van der Waal interactions and no doubt also several C-H$\cdots\pi$ interactions. We thus conclude that PEG binds through a combination of hydrogen bonding with Arg-26, and the formation of various additional interactions with the aromatic residues in P2/P2-lid. This structure thus further underlines the adaptability of the binding site and the versatility of the aromatic residues found there. (E) Structural variation of inward facing occluded $PepT_{St}$. $PepT_{St}$[phosphate] is partially occluded due to the interactions of the PEG molecule with P2-lid. Here an overlay is shown of inward open $PepT_{St}$[apo] (light orange) and all inward facing occluded structures from this study, *i.e.* $PepT_{St}$[phosphate] (pale violet), $PepT_{St}$[Ala-Leu] (salmon) and $PepT_{St}$[Ala-Gln] (mint). Note that the various substrates all interact differently with Trp-427, which correlates with differences in the bending of TM11. The nature of the substrate thus partially dictates the structure of the substrate-bound inward facing occluded form. Adding to this, the stability of this form probably also depends on the nature of the substrate (see main text). The inward facing occluded form is thus not a very well defined state, as also suggested by our previous comparative analysis of several different crystal forms of inward facing $PepT_{St}$ (Quistgaard et al., 2017), but rather a quite variable intermediate between the fully inward open state and an as yet structurally uncharacterized outward facing state, for which the structure and dynamics can be influenced by the bound substrate.
